# 

**Published:** 1916-07-29

**Authors:** 


### HOSPITAL RESULTS IN 1915.

**Ayr County Hospital.**—There were 1,039 in-patients, a decrease of twenty, and 342 out-patients, a decrease of 369. Total income (including legacies £1,400), £4,924; ordinary expenditure, £4,886; extraordinary, £548. Patients' payments amounted to £304.

**Devonshire Hospital, Buxton.**—The hospital is £11,500 in debt, owing to extensions commenced before the war. The illustrations in the report are very interesting, especially those showing the domed central hall and the bath installations. Up to the end of 1915 1,111 soldiers, suffering mostly from rheumatism and allied diseases, had been admitted. The cost of provisions in 1915 was 40 per cent. higher than in the previous year.

**New Hospital for Women, Euston Road. N.W.**—There was unusual pressure upon the in-patient accommodation owing to beds not being available in other hospitals, and to meet the difficulty summer cleaning was reduced to an absolute minimum. The total ordinary income, £7,019, included £2,322 from patients' payments. Legacies produced £5,075. The total expenditure was £9,224.

**Royal Alexandra Infirmary, Paisley.**—"The financial position of the institution is at present satisfactory, and the immediate prospects are not unfavourable." The total income was £9,308 and the expenditure £9,651. The Sir William Dunn Fund, apparently available for current purposes, has investments valued at about £10,000. The accounts are not kept on the Uniform System.

**Royal Free Hospital.**—Of the 2,346 in-patients, 1,213 came from neighbouring districts, 734 from other London districts, and 399 from outside London; 264 soldiers were admitted during the year. Ordinary income, £15,554; legacies, £7,811; total expenditure, £23,966, including £921 for interest. The loans to the hospital amount to £25,000.

RATES OF SUBSCRIPTION: Twelve months, 6/6; Six months, 4/-; Three months, 2/-, post free to any address in the United Kingdom. Abroad, Twelve months, 8/8; Six months, 5/-; Three months, 2/6, post free. THE HOSPITAL "Index" to Volume LIX., October 1915 to March 1916, is now ready, price 1½d. per copy, post free. Address The Manager, "THE HOSPITAL," The Hospital Building, 28/29 Southampton Street, Strand, London, W.C.

### THE BUSINESS OF CHARITY.

**The Standard Technical Book for Chairmen, Governors, Givers, Workers, Matrons, and Secretaries.**

### BURDETT'S HOSPITALS AND CHARITIES

**The Year Book of Philanthropy and Hospital Annual.**

**By Sir HENRY BURDETT, K.C.B., K.C.V.O.**

The 1916 TWENTY-SEVENTH edition contains complete information concerning some 6,000 institutions. Its exhaustive character and completeness have made this book indispensable to all who give with intelligence and guard their gifts with knowledge.

"Approaches . . . the ideal of what a work of reference ought to be." —*The Times.*

"This book is aptly described as a treasure house of information embracing the whole field of charity."—*Morning Post.*

"In its own sphere as well known and as valuable an annual as the other 'Burdett' has long been in the world of finance."—*Truth.*

"We know of no other reference book for hospital charities which contains such a large amount of practical information with regard to hospitals."—*Lancet.*

"Deserves warm commendation, and is a model of wide and accurate presentation of detail."—*Athenæum.*

**NOW READY. Crown 8vo, bound in scarlet cloth over 1,000 pages. 10/6 net. By post, 11/-.**

*Of all Booksellers, or of*

**THE SCIENTIFIC PRESS, LIMITED,**
**28/29 Southampton Street, Strand, London W.C.**

